# Somatic and Stem Cell Bank to study the contribution of African ancestry to dementia: African iPSC Initiative

**DOI:** 10.1002/alz.70145

**Published:** 2025-04-12

**Authors:** Mahmoud B. Maina, Murtala B. Isah, Jacob A. Marsh, Zaid Muhammad, Larema Babazau, Abdulrahman Alkhamis Idris, Ekaterina Aladyeva, Nadia Miller, Emma Starr, Katherine J. Miller, Scott Lee, Miguel Minaya, Selina Wray, Oscar Harari, Baba W. Goni, Louise C. Serpell, Celeste M. Karch

**Affiliations:** ^1^ Biomedical Science Research and Training Centre Yobe State University Damaturu Nigeria; ^2^ Sussex Neuroscience School of Life Sciences University of Sussex Brighton UK; ^3^ Department of Biochemistry Umaru Musa Yar'adua University Katsina Katsina Nigeria; ^4^ Department of Psychiatry Washington University in St. Louis St. Louis Missouri USA; ^5^ Division of Neurogenetics Department of Neurology The Neuroscience Research Institute College of Medicine The Ohio State University Wexner Medical Center Columbus Ohio USA; ^6^ Department of Neurodegenerative Disease UCL Queen Square Institute of Neurology London UK

**Keywords:** African ancestry, Alzheimer's disease, cell bank, clustered regularly interspaced palindromic repeats/CRISPR‐associated protein 9, fibroblasts, frontotemporal dementia, induced pluripotent stem cells, Parkinson's disease, polygenic risk scores

## Abstract

**INTRODUCTION:**

Africa, home to 1.4 billion people and the highest genetic diversity globally, harbors unique genetic variants crucial for understanding complex diseases like neurodegenerative disorders. However, African populations remain underrepresented in induced pluripotent stem cell (iPSC) collections, limiting the exploration of population‐specific disease mechanisms and therapeutic discoveries.

**METHODS:**

To address this gap, we established an open‐access African Somatic and Stem Cell Bank.

**RESULTS:**

In this initial phase, we generated 10 rigorously characterized iPSC lines from fibroblasts representing five Nigerian ethnic groups and both sexes. These lines underwent extensive profiling for pluripotency, genetic stability, differentiation potential, and Alzheimer's disease and Parkinson's disease risk variants. Clustered regularly interspaced palindromic repeats (CRISPR)/CRISPR‐associated protein 9 technology was used to introduce frontotemporal dementia‐associated *MAPT* mutations (P301L and R406W).

**DISCUSSION:**

This collection offers a renewable, genetically diverse resource to investigate disease pathogenicity in African populations, facilitating breakthroughs in neurodegenerative research, drug discovery, and regenerative medicine.

**Highlights:**

- We established an open‐access African Somatic and Stem Cell Bank.
- 10 induced pluripotent stem cell lines from five Nigerian ethnic groups were rigorously characterized.
- Clustered regularly interspaced palindromic repeats (CRISPR)/CRISPR‐associated protein 9 technology was used to introduce frontotemporal dementia‐causing *MAPT* mutations.
- The African Somatic and Stem Cell Bank is a renewable, genetically diverse resource for neurodegenerative research.

## 1 BACKGROUND

Africa, home to approximately 1.4 billion people and > 2000 spoken languages (30% of the world's living languages),^1^ harbors the highest genetic diversity of all continents.^2^ This genetic diversity has profound implications for understanding the genetic basis of complex diseases, including neurodegenerative disorders.^3^ Genetic studies reveal significant population‐specific differences in the frequency and distribution of disease‐associated variants for neurodegenerative diseases (e.g., apolipoprotein E [*APOE*] and *ABCA7*).^4^ These genetic differences contribute to distinct disease susceptibilities and phenotypes that remain largely underexplored in African populations.^5^

Induced pluripotent stem cells (iPSCs) can model genetic contributions to disease and help us understand the influence of specific cell populations to pathologic processes. iPSCs derived from dermal fibroblasts or peripheral blood mononuclear cells retain the donor's genetic background;^6^ have exponential proliferative capacity, making them a renewable resource; and can be engineered using clustered regularly interspaced palindromic repeats (CRISPR) technology. Well‐established protocols allow these iPSCs to be differentiated into diverse brain cell types, making them ideal for studying genetic influences on neurodegenerative diseases at the cellular level.^7^, ^8^, ^9^, ^10^, ^11^ Since the advent of the technology used to generate iPSCs from somatic cells nearly two decades ago,^12^, ^13^ several global initiatives have emerged to support iPSC‐based research in Europe,^14^ North America,^15^ and Asia.^15^, ^16^, ^17^ Despite these advances, many iPSC collections still have significant gaps when it comes to the representation of diverse genetic ancestries.^16^ For example, iPSCs from African populations are rare.^18^, ^19^, ^20^ This underrepresentation severely limits the understanding of diseases and genetic variations pertinent to African populations.^21^

To address this gap, we are building an open‐access African Somatic and Stem Cell Bank as part of our African iPSC Initiative. Here, we describe the initial phase of this effort: 10 fibroblast and iPSC lines representing five Nigerian ethnic groups and both sexes. These lines were rigorously evaluated for pluripotency, genetic stability, and differentiation potential. Genomic profiling was performed in the iPSC for neurodegenerative risk genes, such as *APOE*, and polygenic risk scores (PRSs) were calculated to estimate the overall burden of genetic factors associated with African ancestries in Alzheimer's disease (AD) and other neurodegenerative diseases. Using CRISPR/CRISPR‐associated protein 9 (Cas9) genome editing, we further engineered frontotemporal dementia (FTD)‐causing *MAPT* mutations (P301L and R406W). Cells will be shared via a decentralized biobank at the Biomedical Science Research Training Centre (BioRTC) at Yobe State University, Damaturu, Nigeria, which serves as the primary repository (www.BioRTC.com), as well as the Knight Alzheimer Disease Research Center (Knight ADRC) Biomarker Core at Washington University School of Medicine, USA, and at the Cell Bank at the School of Life Sciences, University of Sussex, UK. These iPSC lines, along with the fibroblasts used to generate them, represent the first dedicated open‐access African Somatic and Stem Cell Bank to facilitate breakthroughs in the neurodegenerative field, drug discovery, and regenerative medicine.

## 2 METHODS

### 2.1 Donor eligibility and consent

Ethical approval for this work was obtained from the Yobe State University Teaching Hospital Ethics Committee (approval reference: YSUTH/MAC/EA/077/VOL.III.297). Healthy adult donors were recruited based on eligibility criteria that required the absence of underlying chronic illnesses and neurological disorders, as well as negative screening results for common transmissible pathogens, including human immunodeficiency virus and hepatitis, to ensure the suitability of the samples for downstream iPSC applications. Before participation, all donors received detailed information about the study, including its purpose, procedures, potential risks, and benefits, both verbally and in writing (see supporting information). Written informed consent was obtained from all participants prior to sample collection. Consent included permission to derive, bank, and distribute iPSC lines for use in future research studies in academia and industry globally. Participants were assured that their data and samples would remain confidential and that their participation was voluntary.

### 2.2 Skin biopsy collection and donor demographics

A qualified surgeon at Yobe State University Teaching Hospital performed the procedure using a small surgical incision. Before the procedure, the forearm area was sterilized, and a local anesthetic (lidocaine) was administered to numb the site. After excision, the tissue samples were immediately rinsed twice in sterile phosphate‐buffered saline (PBS) using two separate sterile 50 mL Falcon tubes to remove any contaminants. The biopsies were then transferred into sterile tubes containing Dulbecco's modified eagle medium (DMEM) supplemented with 1% (v/v) L‐glutamine (L‐Glu), 1% (v/v) penicillin/streptomycin (Pen/Strep), 2.5% (v/v) HEPES solution, and 10% (v/v) fetal calf serum (FCS). The biopsies were subsequently used to establish primary fibroblast cultures.

**FIGURE 1:**
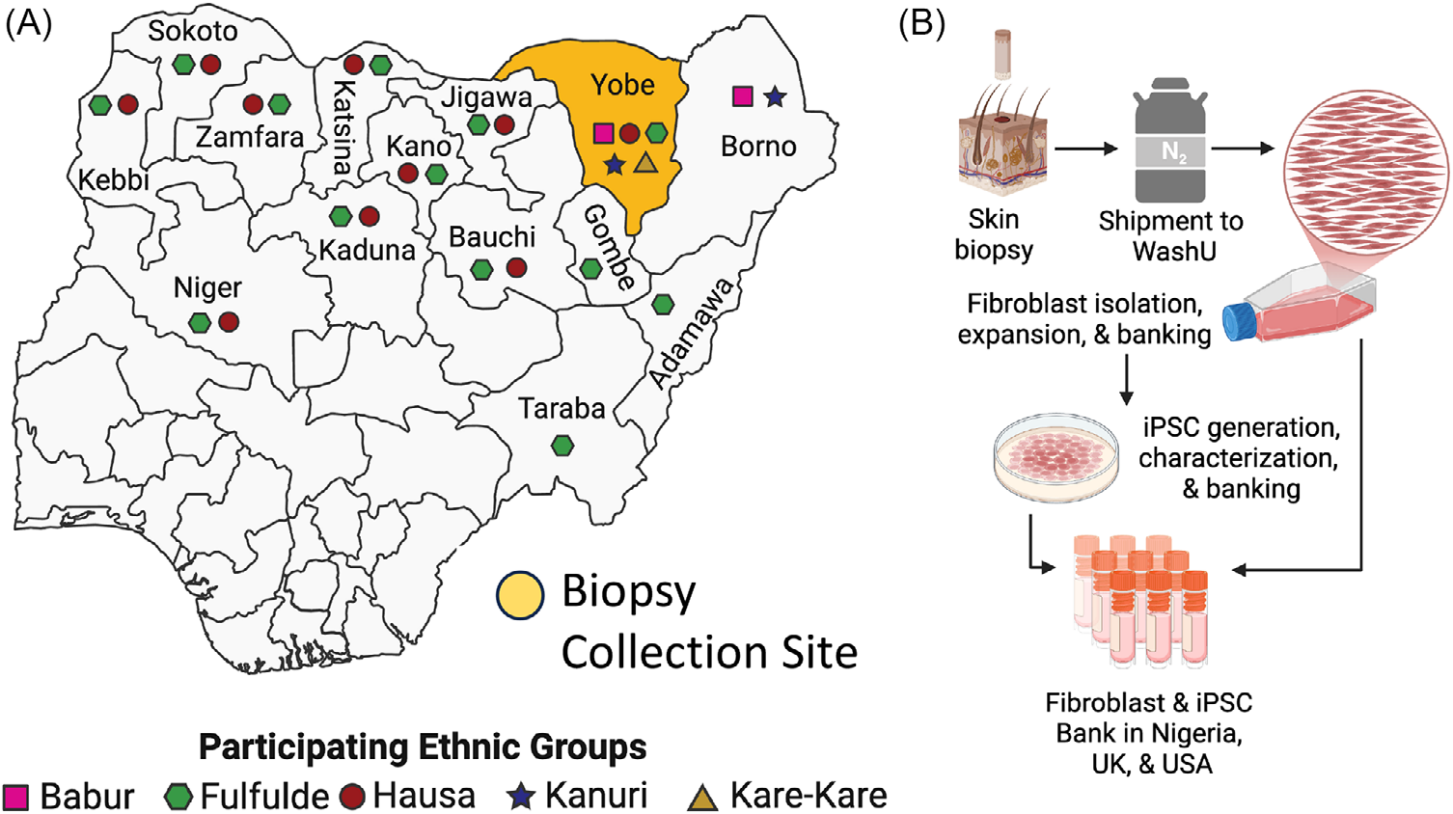
Establishing an African Somatic and Stem Cell Bank. A, Map of Nigeria indicating biopsy collection site (yellow) in the state of Yobe. Ethnic groups participating in the study indicated along with their regional distribution. Pink square, Babur. Green hexagon, Fulfude. Red circle, Hausa. Blue star, Kanuri. Yellow triangle, Kare‐Kare. B, Workflow to transform skin biopsies into fibroblasts and iPSCs. iPSC, induced pluripotent stem cells; WashU, Washington University.

The cohort included 10 donors representing five ethnic groups from Northern Nigeria: Kanuri, Hausa, Babur/Bura, Fulani, and Kare‐Kare (Figure 1). Equal numbers of male and female participants were recruited, ranging from 18 to 60 years (Table 1).

**RESEARCH IN CONTEXT**

1. **Systematic review**: African populations remain underrepresented in induced pluripotent stem cell (iPSC) collections, limiting the exploration of population‐specific disease mechanisms and therapeutic discoveries. To address this gap, we established an open‐access African Somatic and Stem Cell Bank.
2. **Interpretation**: We generated 10 rigorously characterized iPSC lines from fibroblasts representing five Nigerian ethnic groups and both sexes. These lines underwent extensive profiling for pluripotency, genetic stability, differentiation potential, and Alzheimer's disease and Parkinson's disease risk variants. Clustered regularly interspaced palindromic repeats (CRISPR)/CRISPR‐associated protein 9 technology was used to introduce frontotemporal dementia‐associated *MAPT* mutations (P301L and R406W).
3. **Future directions**: This collection offers a renewable, genetically diverse resource to investigate disease pathogenicity in African populations, facilitating breakthroughs in neurodegenerative research, drug discovery, and regenerative medicine.

**TABLE 1:** Human iPSC and fibroblasts.

iPSC line name	Donor fibroblast	Sex	Age at biopsy	Ancestry	*APOE*
BA‐001.1	BA‐001	M	35‐40	Babur	33
BA‐001.2	BA‐001	M	35‐40	Babur	33
BA‐002.1	BA‐002	M	18‐25	Kanuri	23
BA‐002.2	BA‐002	M	18‐25	Kanuri	23
BA‐003.1	BA‐003	M	45‐50	Hausa	23
BA‐003.2	BA‐003	M	45‐50	Hausa	23
BA‐004.1	BA‐004	M	18‐25	Kare Kare	23
BA‐004.2	BA‐004	M	18‐25	Kare Kare	23
BA‐005.1	BA‐005	M	45‐50	Fulani	33
BA‐005.2	BA‐005	M	45‐50	Fulani	33
BA‐006.1	BA‐006	F	60‐65	Kanuri	33
BA‐006.2	BA‐006	F	60‐65	Kanuri	33
BA‐007.1	BA‐007	F	25‐30	Babur	33
BA‐007.2	BA‐007	F	25‐30	Babur	33
BA‐008.1	BA‐008	F	18‐25	Hausa	33
BA‐008.2	BA‐008	F	18‐25	Hausa	33
BA‐009.1	BA‐009	F	18‐25	Fulani	23
BA‐009.2	BA‐009	F	18‐25	Fulani	23
BA‐010.1	BA‐010	F	18‐25	Kare Kare	24
BA‐010.2	BA‐010	F	18‐25	Kare Kare	24

Abbreviations: *APOE*, apolipoprotein E; iPSC, induced pluripotent stem cell.

### 2.3 iPSC generation

Human fibroblasts (Table 1) were transduced with non‐integrating Sendai virus (SeV) carrying OCT3/4, SOX2, KLF4, and cMYC (Life Technologies, A16518) in feeder‐ and serum‐free conditions using mTeSR1 (STEMCell Technologies, 85850) as previously described.^22^ Cells that showed morphological evidence of reprogramming were selected by manual dissection and maintained using mTeSR1. Three stable clones were expanded and banked for each donor line.

### 2.4 iPSC characterization

**FIGURE 2:**
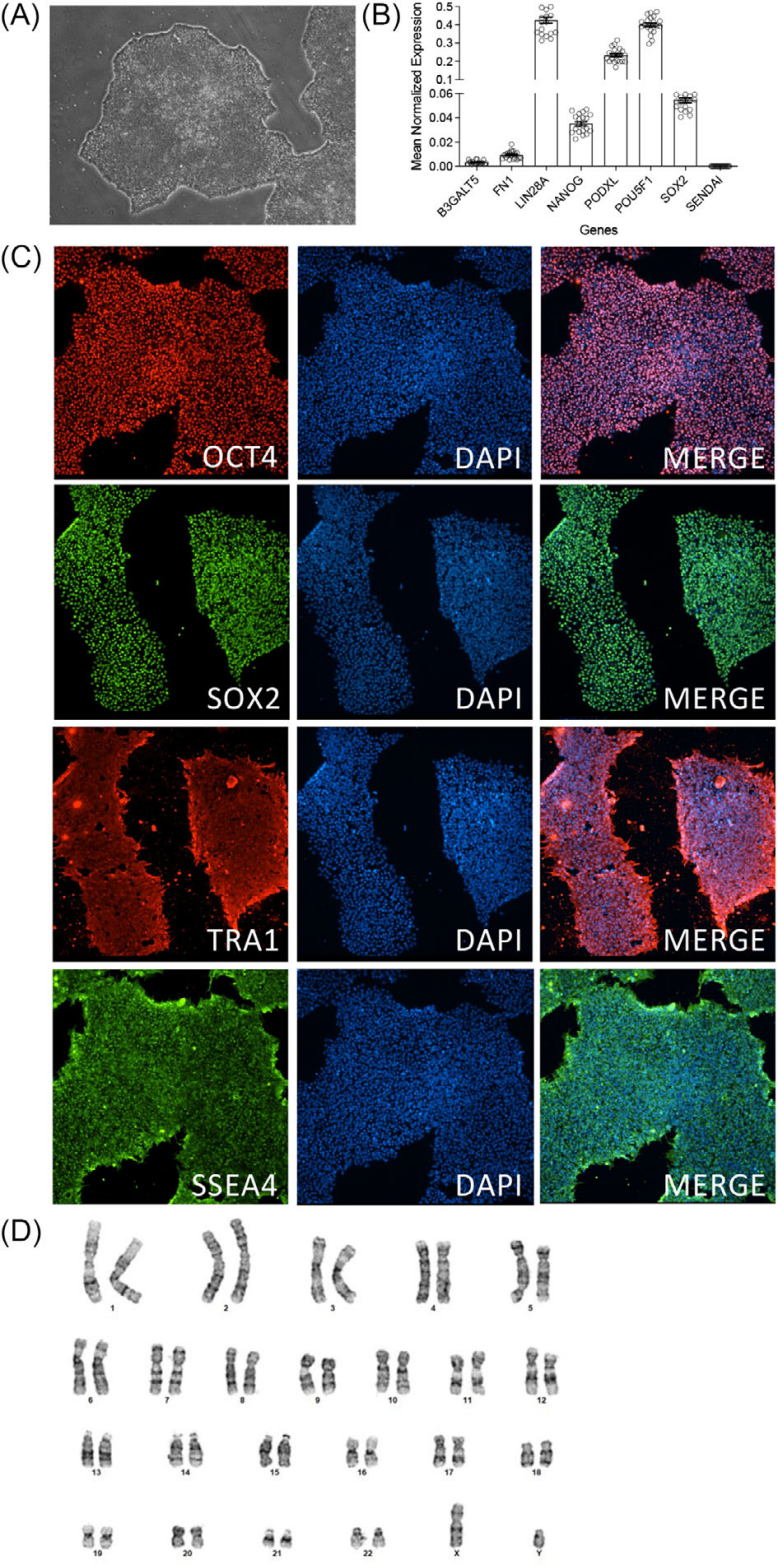
Characterization of iPSC lines from African Somatic and Stem Cell Bank. Representative characterization data. A, Phase‐contrast images of iPSC BA‐001.1. B, qPCR for pluripotency markers normalized against GAPDH for all iPSCs profiled. Open circles represent individual iPSC clones. C, Immunocytochemistry for pluripotency markers OCT4, SOX2, SSEA4, and TRA‐1‐60 in iPSC BA‐001.1. D, G‐band karyotyping reveals no chromosomal abnormalities in iPSC BA‐001.1. See Figures S1–S10 for characterization of all iPSC described in this study. GAPDH, glyceraldehyde 3‐phosphate dehydrogenase; iPSC, induced pluripotent stem cell; qPCR, quantitative polymerase chain reaction.

Human iPSC lines (Table 1) were characterized using standard methods.^22^ Authentication of the iPSC lines to their respective parental fibroblast was completed using short tandem repeat (STR) genotyping (Table S1 in supporting information). Two clones for each donor line were analyzed for pluripotency markers (SOX2, SSEA4, OCT4, TRA‐1‐60) by immunocytochemistry and quantitative polymerase chain reaction (qPCR) and for chromosomal abnormalities by G‐band karyotyping (Figure 2; Figures S1–S10 in supporting information). Cell lines were confirmed to be free of mycoplasma. Detailed methods for each modality are described below.

### 2.5 *APOE* genotyping

Genomic DNA (gDNA) was extracted from cell pellets using a cell lysis solution (0.05% sodium dodecyl sulfate, 0.1 M Tris buffer, 0.1 M ethylenediaminetetraacetic acid [EDTA] in Milli‐Q Water) as well as a protein precipitation solution (8 M ammonium acetate). Cell lysis suspension was centrifuged for 1 minute at 16000 x g. Supernatant was transferred to a solution of 100% isopropanol to isolate gDNA. gDNA pellets were washed with 70% ethanol and dissolved in 1X Tris‐EDTA (TE) Buffer (G Biosciences, R025). *APOE* genotyping was completed by qPCR using an ABI Real Time Taqman SNP Genotyping assay. TaqMan probes used for allelic determination included rs429358 (C_3084793_20) and rs7412 (C_904973_10). *APOE* genotypes were defined by combining the results for both rs429358 and rs7412: *APOE* ε2 (rs429358 [T] and rs7412 [T]); *APOE* ε3 (rs429358 [T] and rs7412 [C]); *APOE* ε4 (rs429358 [C] and rs7412 [C]).

### 2.6 iPSC culture

Human iPSCs were cultured in mTeSR1 on Cultrex Basement Membrane Extract (BME)‐coated tissue culture‐treated plates (R&D Systems, 3532‐010‐02). For routine passaging and unless otherwise noted below, iPSCs were dissociated with Accutase (STEMCell Technologies, 07922) for 3 minutes. Dissociated cells were collected in PBS and centrifuged at 131×g for 3 minutes. After medium was aspirated, the cells were plated on new BME‐coated plates in mTeSR1. iPSCs were maintained with < 5% spontaneous differentiation, based on morphology evaluated by bright field microscopy, and cryopreserved in mTeSR1 supplemented with 10% dimethyl sulfoxide and 40% fetal bovine serum (FBS). iPSCs are karyotyped every 10 passages per International Society for Stem Cell Research guidelines to ensure clones maintain stable genomes. All cell lines were confirmed to be mycoplasma‐free using the MycoAlert mycoplasma detection kit (Lonza, LT07‐710) according to the manufacturer's instructions.

### 2.7 iPSC genomic editing

Human iPSCs underwent genomic editing via ribonucleoprotein (RNP). Three million cells were prepared by applying Accutase for 10 minutes to achieve dissociation into single cells. Cells were then exposed to single nucleotide polymorphism (SNP)–specific CRISPR RNA and trans‐activating CRISPR RNA at a final concentration of 100 µM, RNP Cas9 at a final concentration of 6.4 µg/µL, and a donor oligonucleotide at a final concentration of 100 µM. Cells then underwent electroporation from Lonza 4D‐Nucleofector X unit and were plated in DMEM supplemented with 10% FBS. Five days post‐nucleofection, cells were serial diluted and picked upon reaching 50 to 100 µm in size. Cells were then screened via next generation sequencing (NGS) for introduction of the SNP of interest (*MAPT* P301L or *MAPT* R406W). iPSC lines that have been exposed to the editing pipeline without a resulting edit are available as “unedited” controls.

### 2.8 Immunocytochemistry

Cells were washed and fixed with 4% paraformaldehyde (Sigma‐Aldrich). Primary and secondary antibodies were diluted in 3% bovine serum albumin. The following antibodies were used: SOX2 (1:500; Cell Signaling Technologies, 3579), SSEA4 (1:500; Cell Signaling Technologies, 4755), TRA‐1‐60 (1:1000; Cell Signaling Technologies, 4746), OCT4 (1:500; Cell Signaling Technologies, 2840), Donkey Anti‐Rabbit Alexa Fluor 594 (1:250; Life Technologies, A‐21207), Goat Anti‐Mouse Alexa Fluor 488 (1:250; Life Technologies, A‐10680), Donkey Anti‐Rabbit Alexa Fluor 488 (1:250; Life Technologies, A‐21206), and Goat Anti‐Mouse Alexa Fluor 594 (1:250; Life Technologies, A‐11005). Nuclei were counterstained with 4′,6‐diamidino‐2‐phenylindole (DAPI; Life Technologies). Images were acquired on a Nikon Eclipse 80i fluorescence microscope (Nikon Instruments) using LMS software.

### 2.9 qPCR

RNA was extracted from cell pellets with an RNeasy kit (QIAGEN, 74106), following the manufacturer's protocol. Extracted RNA (10 µg) was converted to complementary DNA (cDNA) by PCR using the High‐Capacity cDNA Reverse Transcriptase Kit (Life Technologies, 4368813). Gene expression was measured in iPSCs for *SOX2*, *POU5F1*, *LIN28A*, *NANOG*, *B3GALT5*, and *PODXL* using qPCR and normalized against glyceraldehyde 3‐phosphate dehydrogenase (GAPDH) as previously described.^8^ Primers specific to SeV were included to evaluate the presence of virus remaining in the isolated clones. Primers specific to *GAPDH* were used as a control.

### 2.10 Karyotyping

Chromosomal abnormalities were assessed by G‐band karyotyping.

### 2.11 Trilineage differentiation

The capacity to form cell types from the three germ layers was confirmed by differentiating cells using STEMdiff Trilineage Differentiation Kit (STEMCell Technologies, 05230). Briefly, cells were plated according to manufacturer's protocol and supplemented with either STEMdiff Trilineage Endoderm Medium, STEMdiff Trilineage Ectoderm Medium, or STEMdiff Trilineage Mesoderm Medium. Cells were harvested after 5 days, and qPCR was performed as previously described to assess expression of germ layer–specific markers (ectodermal markers: *TUBB3* and *Pax6*; endoderm markers: *FOXA2* and *CXCR4*; mesodermal markers: *Desmin* and *SMA*). Primers specific to *GAPDH* and *Fibronectin* were used as endogenous controls and fibroblast markers, respectively.

### 2.12 Genomic analyses

gDNA was isolated as described above and resuspended at a dilution of at least 50 ngl/ul. Samples were analyzed by an Infinium Global Screening Array‐24 v.30 GWAS Chip. Quality control (QC) of the genome‐wide association study (GWAS) data was performed using PLINK 1.9^23^ and plinkQC 0.3.4.^24^ Low‐quality SNPs were filtered using the following criteria: (1) SNP missing rate > 1%, (2) minor allele frequency (MAF)  < 5%, and (3) Hardy–Weinberg *P* value  < 1 × 10^−6^ (Table S2 in supporting information). Samples were verified for sex inconsistencies by matching phenotypic data with genetic sex imputation from the X‐chromosomal heterozygosity rate. An identity‐by‐descent (IBD) analysis was performed to identify pairs of fibroblasts and iPSC lines from the same donor and to estimate the genetic relationships between individuals. SNPs from *APOE* genotyping (rs7412 and rs429358) were added manually to the GWAS results.

After QC, bcftools 1.21^25^ was used to convert SNP files to VCF files, following the data phasing with Eagle v2.4^26^ and the imputation of un‐genotyped variants using Michigan Imputation Server 2 (version 2.0.6)^27^ with 1000 Genomes Phase 3 v5 as a reference panel.^28^ Imputed SNPs with *R* ^2^ < 0.3 were removed and used to estimate the global ancestry of samples by running principal component analysis (PCA) on 1000 Genomes Phase 3 v5 reference data and projecting the calculated principal components to the target dataset using default parameters. The FRAPOSA tool^29^ was used to estimate the distance to the population in the reference panel.

### 2.13 PRS

PRS for AD and Parkinson's disease (PD) were calculated with pgsc_calc pipeline.^30^, ^31^, ^32^, ^33^ Score files with GWAS risk estimates were downloaded from the PGS catalog.^30^ GWAS summary statistics were used as a reference for AD and PD polygenic score (PGS) calculations. PGS003956^34^ includes variants associated with AD risk within individuals of African ancestry,^35^ and PGS000903^36^ summarizes the genetic risk for PD in a multi‐ancestry cohort. A comparison dataset of African population genotypes was obtained from 1000 Genomes Phase 3 v5 data by selecting donors with African global ancestry. PRS excluding *APOE* regions were calculated separately by excluding SNPs within the 200Kb region surrounding the *APOE* gene locus (hg19 positions—chr19:45,211,000‐45,613,000).

### 2.14 Local ancestry inference

Local ancestry was inferred for individual chromosomes using FLARE (version 0.5.2).^37^ This method allows for investigation of the ancestral origin of an allele using phased and imputed GWAS data. A reference panel was obtained from 1000 Genomes Phase 3 v5. We used common SNPs that were called in our filtered dataset and in the reference dataset. Local ancestry was inferred for individual chromosomes. Default values were used for all parameters, except calculation of the posterior ancestry probabilities (i.e., –probs). The random seed was specified for the reproducibility of analysis (–seed).

### 2.15 RNA sequencing

iPSCs were resuspended in 200 µL of 50:1 homogenization solution: 1‐thioglycerol solution. After the addition of 200 µL of Promega lysis buffer, the samples were transferred to the appropriate well of the Maxwell RSC cartridge. DNase solution was added to each cartridge. TapeStation 4200 System (Agilent Technologies) was used to perform QC of the RNA concentration, purity, and degradation based on the estimated RNA integrity number (RIN) and DV200. Samples were sequenced by an Illumina HiSeq 4000 Systems Technology with a read length of 1 × 150 bp and an average library size of 36.5 ± 12.2 million reads per sample.

IBD^38^ and FastQC^39^ analyses were performed to confirm sample identity. STAR (v.2.6.0)^40^ was used to align the RNA sequences to the human reference genome: GRCh38.p13 (hg38). The quality of RNA alignment was evaluated using sequencing metrics such as read distribution, ribosomal content, and alignment quality in Picard (v.2.8.2).

Salmon (v. 0.11.3)^41^ was used to quantify the expression of the genes annotated within the human reference genome used in this project (GRCh38.p13). Protein coding genes were selected for downstream analyses. PCA was calculated based on the 500 most variably expressed genes obtained from 19,957 protein‐coding genes using regularized logarithm transformation (rlog) counts. PCA and histogram plots were created using the ggplot2 package (v.3.3.6).^42^

## 3 RESULTS

### 3.1 Community engagement and development of the African Somatic and Stem Cell Bank

Africa's unparalleled genetic diversity is vastly underrepresented in current cellular models.^21^ We set out to address this critical gap by building an open‐access African Somatic and Stem Cell Bank. Cells in this bank represent indigenous Africans (AFR) from Nigeria, where we have leveraged decades‐long connections and collaboration with communities to facilitate engagement with this initiative.^43^, ^44^, ^45^

To achieve broad community participation, we conducted extensive outreach activities, including radio campaigns, across northeastern Nigeria to raise awareness of the study and recruit participants. Informed consent was secured from healthy donors who met eligibility criteria (see section 2). Skin biopsies were performed on 10 donors (5 males and 5 females) representing five ethnic groups in the region: Kanuri, Hausa, Babur/Bura, Fulani, and Kare‐Kare (Figure 1; Table 1). Each sample was assigned a unique identifier to maintain anonymity. Donors were eligible if both parents belonged to the same ethnic group, ensuring consistency in self‐reported ethnicity. However, we recognize that historical and generational genetic admixture may exist beyond immediate parental lineage. The ethnic donors recruited in the Yobe State are broadly represented across northern Nigeria and, in some cases, extend beyond Nigeria's borders (Figure 1A). The Hausa and Fulani populations are widespread across West Africa, while the Kanuri population has historical ties to the Kanem–Bornu Empire, with communities spanning regions of Cameroon, Niger, and Chad.

Skin biopsies were processed to obtain primary fibroblast cultures and banked to ensure accessibility for future research. This initial cohort is primarily composed of young individuals without incidence of neurodegenerative disease at the time of sampling. While systematic family history assessments were not conducted, donors were asked about any personal history of neurological disorders during recruitment, and only those without such a history were included. Donor information has been securely recorded, and permission for follow‐up was obtained, allowing for the possibility of recontacting donors in future studies to assess their neurological health status over time. To further strengthen this resource for dementia research, we are now developing a dedicated cohort in northern Nigeria that includes individuals with AD alongside age‐ and sex‐matched controls. This cohort will undergo comprehensive clinical and genetic characterization to enhance its utility for studying dementia risk factors, mechanisms, and disease progression. The collection of fibroblast and erythroid progenitor cells from the cohort will continue to expand the somatic cell bank. Together, these efforts represent a pivotal step in the African iPSC Initiative: fostering community participation and trust while establishing a somatic cell resource that will support future scientific discoveries and global research collaborations.

### 3.2 Generation of iPSCs

Fibroblast cultures from the 10 donor lines were reprogrammed into iPSCs using non‐integrating SeV vectors carrying OCT3/4, SOX2, KLF4, and cMYC (Figure 1B; Table 1). Phase‐contrast microscopy revealed that colonies from all the iPSCs have a high nuclear‐to‐cytoplasmic ratio with prominent nucleoli, grow in colonies, and have well‐defined borders, characteristic of iPSCs (Figure 2A). RNA expression of key pluripotency markers, including SOX2, POU5F1, LIN28A, NANOG, and PODXL, were increased (Figure 2B, Figures S1–S10). Expression levels of pluripotency markers were similar across iPSC donor lines (Figure 2B; Figures S1–S10). Stability of iPSC clones depends upon expression of endogenous pluripotent genes and elimination of residual exogenous SeV. Primers specific to SeV illustrated clearance of residual virus in the reprogrammed clones (Figure 2B; Figures S1–S10). Immunostaining with antibodies for the pluripotency markers SOX2, OCT4, SSEA4, and TRA‐1‐60, further confirmed the pluripotent identity of the iPSC lines (Figure 2C; Figures S1–S10).

To evaluate the genomic integrity of the iPSC lines, karyotyping analysis was conducted, revealing no detectable chromosomal abnormalities (Figure 2D; Figures S1–S10). STR profiling illustrates that all the iPSC clones are a genetic match for their parental fibroblast lines (Table S1). Together, these analyses demonstrate that the iPSC lines are stable and robust for downstream applications.

### 3.3 Genomic and molecular characterization of iPSCs

**FIGURE 3:**
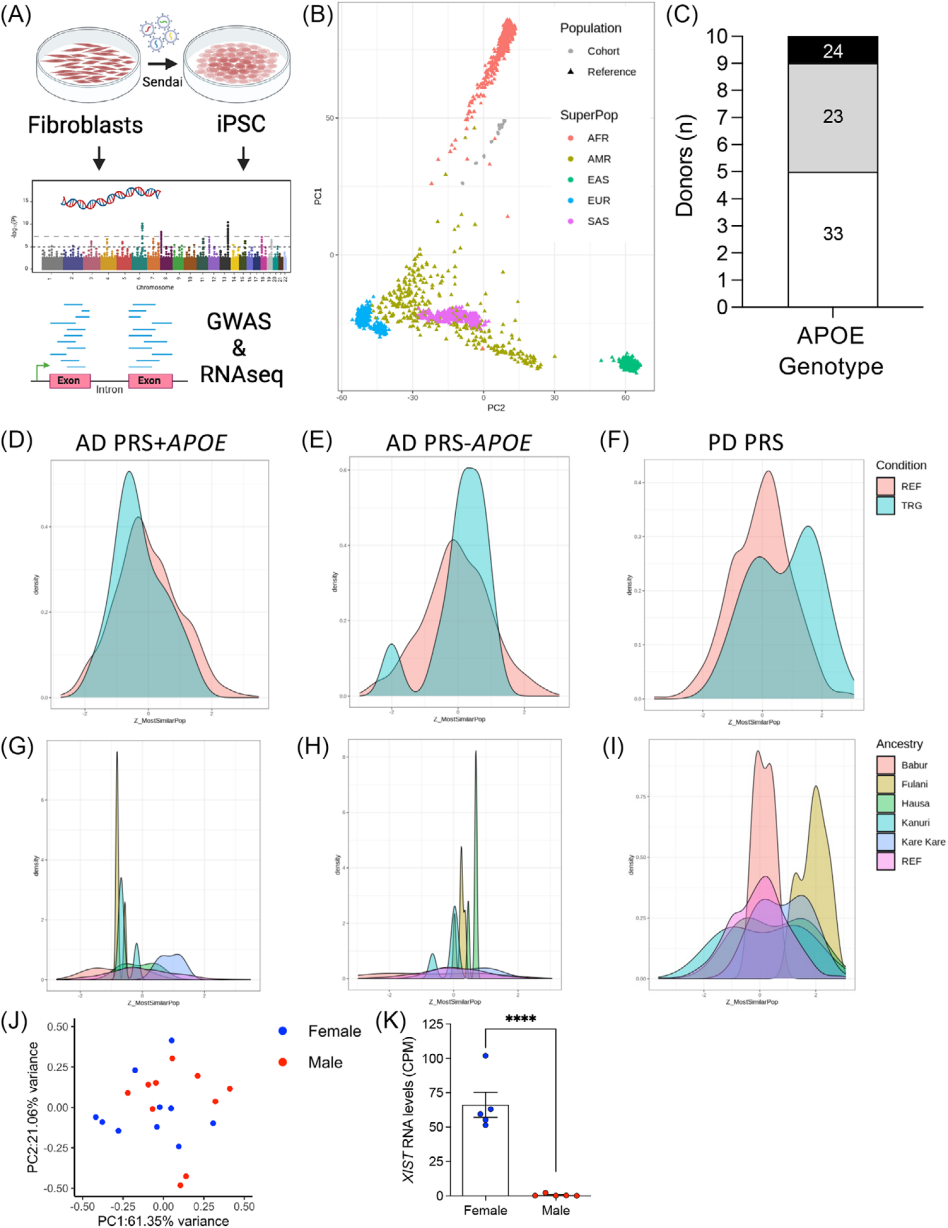
Genomic profiling of iPSC lines from African Somatic and Stem Cell Bank. A, Workflow. B–I, Genetic analyses of iPSC. B, PCA of common genetic variants measured in unique iPSC lines (gray, cohort) compared to 1000 Genomes reference: AFR, AMR, EAS, EUR, SAS. C, Frequency of APOE genotypes among iPSC lines. D–I, PRS. D–E, Distribution of PRS represented using reference (salmon, REF) and iPSC samples (cyan, TRG). G–H, Distribution of PRS represented based on Nigerian ethnic group. D, G, PRS for AD including the APOE gene locus. E, H, PRS for AD excluding the APOE gene locus. F, I, PRS for PD. J, PCA of transcriptomic data generated from iPSCs (500 most variable gene transcripts). K, XIST, expressed on the inactivated X‐chromosome, in iPSC lines. Graphs represent CPM mean ± SEM. ****, *p* < 0.0001. Blue, female. Red, male. AD, Alzheimer's disease; AFR, Africans; AMR, admixed Americans; *APOE*, apolipoprotein E; CPM, counts per million; EAS, East Asians; EUR, Europeans; GWAS, genome‐wide association study; iPSC, induced pluripotent stem cell; PCA, principal component analysis; PD, Parkinson's disease; PRS, polygenic risk score; REF, reference; SAS, South Asian; SEM, standard error of the mean.

To maximize utility of the cell bank, genomic analyses were performed to evaluate common variants (Figure 3A). After QC (see section 2), genotype information from 333,080 SNPs was used to evaluate genetic ancestry of the iPSC lines. PCA was performed by comparing each iPSC line to the 1000 Genomes reference ancestry panels. PCA revealed that the iPSC lines (gray circles) cluster closest to the reference data from African/African American ancestry (Figure 3B). Thus, the genomic background of the iPSC lines are enriched for African/African American ancestry.

The three major *APOE* isoforms*, APOE* ε2*, APOE* ε3, and *APOE* ε4, contribute to AD risk to varying degrees*. APOE* ε4 has been identified as a major risk factor for AD, while *APOE* ε2 is considered protective.^46^ *APOE* ε3/ε3 was the most common genotype among the cell lines (*n* = 5, Figure 3C), which aligns with previous studies reporting *APOE* ε3/ε3 as the most common genotype among Yorubas in Nigeria.^47^ While the frequency of *APOE* ε2/ε3 is higher than we would expect within European datasets (*n* = 4), this is consistent with *APOE* genotype distribution in Nigerian cohorts (12%–15.9%).^47^, ^48^ However, there are currently limited data on *APOE* distribution stratified by specific African ethnic groups, highlighting the need for further research in diverse populations. For AD, *APOE* ε3/ε3 is considered neutral across ancestral backgrounds.^46^ *APOE* ε2/ε3 (*n* = 4) is associated with reduced AD risk (odds ratio [OR] 0.6), and *APOE* ε2/ε4 (*n* = 1) is associated with increased AD risk (OR 2.6; Figure 3C).^49^ Of note, the penetrance of *APOE* genotypes differs across ancestral backgrounds.^50^ *APOE* ε4 has a lower impact on AD risk when it occurs in a genetic background with African ancestry (OR 2.1 compared to OR 7.2 in European ancestry backgrounds).^35^, ^50^, ^51^ While the reason for these differences remains unclear, they may be driven by additional variants in the *APOE* haplotype that modify the effects of *APOE* ε4, differences in chromatin accessibility in the *APOE* locus, or by other mechanisms.^51^, ^52^, ^53^ We calculated global and local ancestry of each chromosome using FLARE (see section 2). This approach predicts a high probability of African ancestry at each chromosome, as we expected (Table S3 in supporting information). Analyses of local ancestry around the *APOE* locus confirms all lines are 100% African ancestry (Table S3). Thus, the iPSC collection represents *APOE* genotypes that capture the spectrum of AD risk.

While *APOE* is a major risk factor for AD, additional common variants contribute to disease risk.^46^ The majority of these variants have small effects on disease risk, and thus, genetic risk profiles are optimally interpreted by combining multiple risk variants to generate a score.^54^, ^55^, ^56^ PRSs are informative for a number of neurodegenerative diseases for which complex genetic architecture underlies risk for sporadic disease. Leveraging genotype data from the 333,080 SNPs, we generated PRSs for each of the lines using the PGS catalogue. In the case of AD, we used the summarized genetic variants associated with AD risk within individuals of African ancestry (Figure 3D–I, Table S4 in supporting information).^30^, ^35^ Distribution of PRS was plotted relative to a reference dataset (donors with African global ancestry in 1000 Genomes Phase 3 v5; Figure 3D–I). PRSs constructed from the summary statistics of five AD GWAS^34^ were plotted for the iPSC lines, including the *APOE* locus (Figure 3D, Table S4) and excluding the *APOE* locus (Figure 3E, Table S4). PRSs were normally distributed, suggesting this collection captures the spectrum of AD PRSs. Additionally, PRSs were calculated using the summary statistics from 17 GWAS for PD^36^ and plotted for the iPSC lines (Figure 3F). PD PRSs were bimodally distributed among the iPSC (Figure 3F, Table S4). As genetic ancestry contributes to common variants that confer disease risk and, in turn, PRS, we sought to evaluate the distribution of PRSs for AD and PD based on the ethnic group of the iPSC (Figure 3G–I). Frequency of high and low PRSs differ based on the ethnic group; however, because the sample numbers remain small, we cannot make biological conclusions about these observations. Together, the sequencing data described here makes each iPSC line a rich genetic resource for investigators who are interested in exploring specific genes/variants of interest.

Molecular profiling of the iPSC lines using bulk RNA sequencing was also performed. By comparing the 500 most variably expressed genes, we find that the iPSCs are distributed in a pattern consistent with prior observations that donor background is the largest contributor to transcriptomic variability.^57^ Furthermore, while transcriptomic profiles do not differ based on sex (Figure 3J), we detected consistent and robust expression of the lncRNA *XIST* in female iPSC lines, which is turned on in the X‐inactivated chromosome (Figure 3K).^58^ Together, the genomic and molecular profiles provide a strong foundation for selection and use of these lines for future studies.

### 3.4 Differentiation potential of the iPSCs

A key component of a high‐quality iPSC line is its capacity to differentiate into cells within the three major germ layers. To evaluate the differentiation capacity of the newly generated iPSC lines, we performed trilineage differentiation assays using the STEMdiff Trilineage Differentiation Kit. Expression analysis revealed robust expression of markers in each of the three lineages. Expression of *PAX6* and *TUBB3*, markers associated with neuroectodermal lineages, confirms ectodermal differentiation (Figures S1–S10). Mesodermal differentiation was validated by the strong expression of *SMA* and *DES*, albeit to a lesser extent (Figures S1–S10). Endodermal differentiation was confirmed with expression of *FOX2A* and *CXCR4* (Figures S1–S10). All 20 iPSC clones (from 10 donor lines) successfully differentiated into cells in each of the three germ layers. Together, we demonstrate that all the iPSC lines can differentiate into derivatives of the three germ layers, highlighting their utility for broad applications.

### 3.5 Genome engineering to model genetic causes of FTD in diverse backgrounds

**FIGURE 4:**
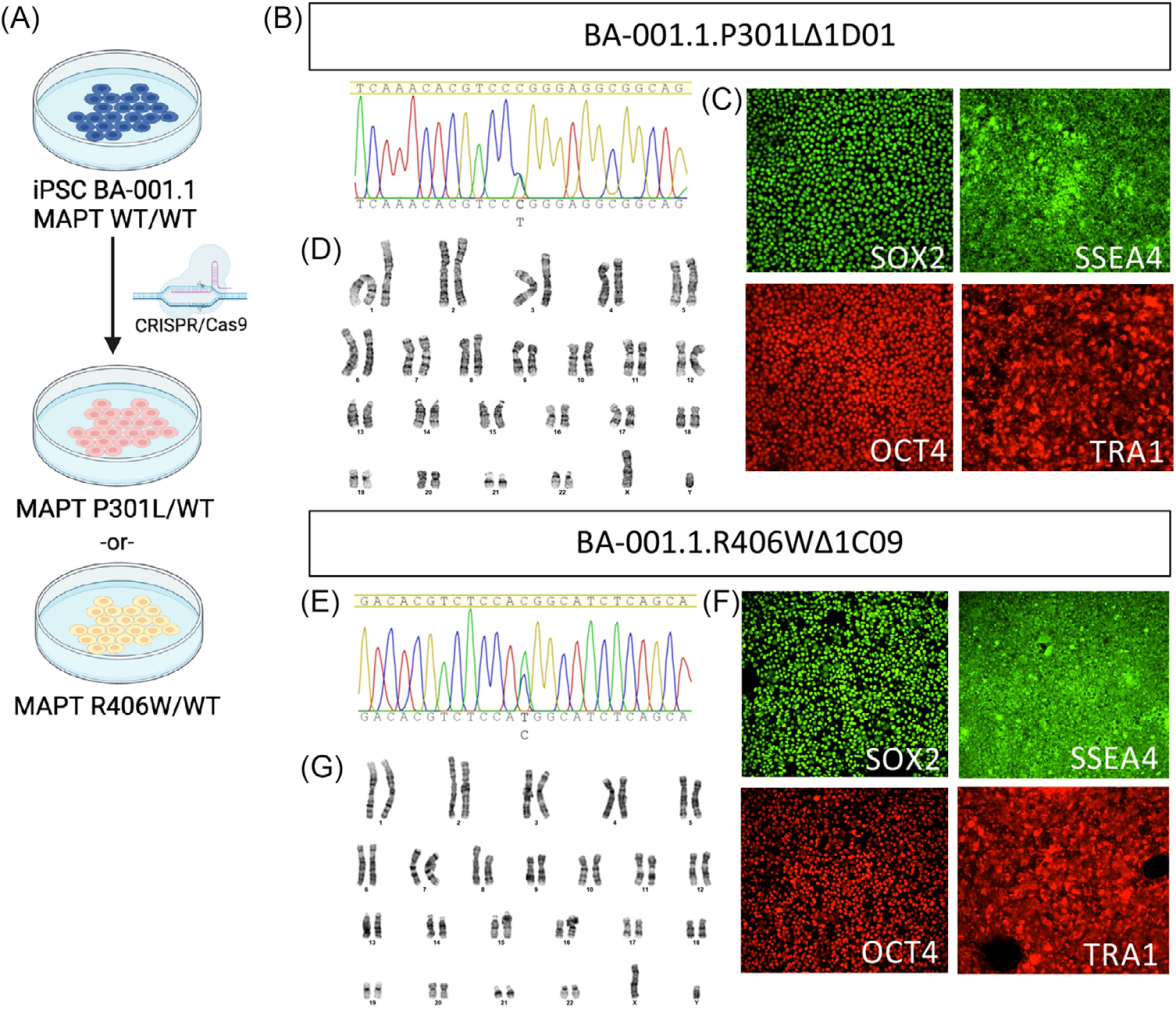
Genome engineering of the *MAPT* locus in iPSC BA‐001.1. A, Workflow. B–D, BA‐001.1 was edited from *MAPT* WT/WT to *MAPT* P301L/WT using CRISPR/Cas9. Characterization for one of the resulting clones: BA‐001.1.P301L Δ1D01. B, Sanger sequencing illustrating the heterozygous engineered SNP. C, Immunocytochemistry for pluripotency markers OCT4, SOX2, SSEA4, and TRA‐1‐60. D, G‐band karyotyping reveals no chromosomal abnormalities. E, F, BA‐001.1 was edited from *MAPT* WT/WT to *MAPT* R406W/WT using CRISPR/Cas9. Characterization for one of the resulting clones: BA‐001.1.R406W Δ1C09. E, Sanger sequencing illustrating the heterozygous engineered SNP. F, Immunocytochemistry for pluripotency markers OCT4, SOX2, SSEA4, and TRA1. CRISPR/Cas9, Clustered regularly interspaced palindromic repeats/CRISPR‐associated protein 9; G, G‐band karyotyping reveals no chromosomal abnormalities; iPSC, induced pluripotent stem cell; SNP, single nucleotide polymorphism.

To begin to expand the utility of these iPSC lines for researchers in the dementia fields, we used CRISPR/Cas9‐mediated genome editing to introduce pathogenic *MAPT* mutations into the newly characterized iPSC lines (Figure 4A; Table 2). This effort aligns with prior endeavors to establish genetically engineered iPSC models for studying tau‐related neurodegeneration.^8^, ^11^ Local ancestry within the *MAPT* locus is 100% African across all lines (Table S3). The iPSC line BA‐001.1, of Babur ancestry, was selected to introduce one of two well‐characterized *MAPT* mutations—P301L or R406W—both of which are associated with frontotemporal lobar degeneration and other tauopathies.^59^, ^60^, ^61^, ^62^ The *MAPT* P301L mutation is one of the most common variants associated with FTD worldwide, leading to behavioral disturbances, aphasia, cognitive impairment, and parkinsonism.^63^ Similarly, the *MAPT* R406W mutation has been identified in multiple families worldwide. Interestingly, *MAPT* R406W carriers exhibit a clinical phenotype that mimics AD, including progressive cognitive impairment.^64^, ^65^, ^66^ These specific *MAPT* mutations are well represented in research studies such as GENFI and ALLFTD. Furthermore, we selected these *MAPT* mutations with the goal of complementing existing patient and engineered lines that carry these mutations in iPSC generated from donors of European ancestry. Cellular phenotypes occurring as a function of these specific mutations have been previously reported.^67^, ^68^, ^69^, ^70^

**TABLE 2:** Genome editing of *MAPT* locus to model frontotemporal dementia.

iPSC	iPSC donor line	Donor fibroblast	Engineered genotype	Sex
BA‐001.1.P301LΔ1D01	BA‐001.1	BA‐001	MAPT P301L/WT	M
BA‐001.1.R406WΔ1C09	BA‐001.1	BA‐001	MAPT R406W/WT	M

Abbreviation: iPSC, induced pluripotent stem cell.

CRISPR/Cas9 editing was performed with reagents previously described for engineering *MAPT* mutations on a wild‐type background.^8^ Two clones were fully characterized for each *MAPT* mutation (Figure 4 and Figures S11–S12 in supporting information). Sanger sequencing chromatograms confirmed heterozygous single‐nucleotide substitutions at the expected *MAPT* loci for both the P301L (Figure 4B; BA‐001.1.P301LΔ1D01) and R406W mutations (Figure 4E; BA‐001.1.R406WΔ1C09). The guideRNAs used for engineering were predicted to be very specific (Table S5 in supporting information). Off‐target sequencing confirmed that the predicted off‐target sites remained identical to the parental line (Table S6 in supporting information). We next evaluated the pluripotency and genomic stability of the edited lines using immunocytochemistry and karyotyping. iPSC BA‐001.1.P301LΔ1D01 and iPSC BA‐001.1.R406WΔ1C09 illustrated robust expression of key pluripotency markers, SOX2, SSEA4, OCT4, and TRA‐1‐60 (Figure 4C,F), consistent with the unedited iPSCs. Additionally, G‐band karyotyping analysis revealed no detectable chromosomal abnormalities in the edited clones, demonstrating that genomic integrity was preserved following the editing process (Figure 4D,G). Together, this mutant *MAPT* engineered series will be a valuable tool for elucidating the molecular mechanisms driving neurodegenerative tauopathies within the unique genetic context of African ancestry. Our long‐term goal remains to introduce these *MAPT* mutations into additional iPSCs within this cohort.

### 3.6 Accessing data and cells from the African Somatic and Stem Cell Bank

To facilitate global access to the fibroblast and stem cell resources, we have implemented a decentralized biobanking system for cell distribution. The cells described here are stored at the BioRTC at Yobe State University, Damaturu, Nigeria, which serves as the primary repository (www.BioRTC.com). To promote global distribution, cells are also stored at the Knight ADRC Biomarker Core at Washington University School of Medicine, USA, and at the Cell Bank at the School of Life Sciences, University of Sussex, UK. Requests for cell lines are managed centrally via the primary repository at BioRTC (https://biortc.com/african‐ipsc‐initiative/). Approved requests require a fully executed universal material transfer agreement (MTA) and will be fulfilled by the repository geographically nearest to the requester to minimize shipping times. Cell lines are available to both non‐profit and for‐profit entities and may be used for applications such as disease modeling and drug screening. However, their use for commercial product development requires prior approval. Our initiative will support diverse research fields, foster global collaboration, and accelerate innovation in understanding and treating human diseases.

## 4 DISCUSSION

Here, we describe the generation of 10 deeply phenotyped iPSC lines from five ethnic groups of northern Nigeria as the initial phase of the African iPSC Initiative to derive and distribute iPSC models from indigenous Africans to the international scientific community. Africans represent the most genetically diverse population globally, harboring a vast array of common variants considered rare in other populations.^2^, ^28^ This genetic diversity presents a rich resource for studying disease mechanisms, drug discovery, and regenerative therapies.^71^, ^72^ Yet, cellular models that capture this diversity are rare.^16^, ^21^ The collection described here builds upon our recent report of the first iPSC line from indigenous Nigerians.^19^

### 4.1 Open access African Somatic and Stem Cell Bank

The availability of population‐specific somatic cells and iPSCs is critical for elucidating the molecular underpinnings of diseases across diverse populations. Globally, major initiatives have emerged to generate and bank iPSCs. For example, in Europe, the European Bank for Induced Pluripotent Stem Cells (EBiSC) serves as a centralized repository for standardized iPSC lines.^14^ In the United States, institutions such as the California Institute for Regenerative Medicine (CIRM), the Coriell Institute for Medical Research, the WiCell Research Institute, the National Centralized Repository for Alzheimer's Disease and Related Dementias (NCRAD), and the NIH Intramural Center for Alzheimer's and Related Dementias (CARD) iPSC Neurodegenerative Disease Initiative (iNDI) via The Jackson Laboratory play key roles in distributing well‐characterized iPSC lines, particularly for neurodegenerative disease research.^15^, ^73^, ^74^ In Asia, the RIKEN BioResource Research Center and Center for iPS Cell Research and Application (CiRA) in Japan, National Stem Cell Bank of Korea, and Human Disease iPSC Consortium Resource Center play key roles in distributing iPSC lines.^15^, ^16^, ^17^ However, among the iPSC lines with assigned ethnicities in these repositories, only a small percentage represent Black or African ancestry, primarily derived from African American donors.^21^ Populations of African descent living outside the continent do not fully capture the immense genetic diversity of the ethnolinguistic groups across Africa.^21^, ^71^, ^72^, ^75^

To address this shortcoming, we have established the first African Somatic and Stem Cell Bank, which contains a collection of somatic cells and iPSC lines. Each cell line in this resource has undergone rigorous QC, including pluripotency testing, karyotyping, and STR profiling to confirm iPSC identity, genetic integrity, and stability. These iPSCs exhibit robust differentiation potential into derivatives of the three germ layers, demonstrating their versatility for diverse applications. These iPSCs can support a broad range of research applications, including disease modeling, drug discovery, and regenerative medicine, thus enabling breakthroughs that better reflect the genetic backgrounds of African populations.

The fibroblasts in our biobank offer unique advantages for translational research. Fibroblasts can be used as primary cells to evaluate cellular phenotypes. For example, in PD, fibroblasts can be used to evaluate α‐synuclein inclusions.^76^ High content microscopy along with AI may reveal disease signatures in fibroblast cells themselves. Additionally, fibroblasts can be directly reprogrammed into induced neurons without transitioning through an iPSC state.^77^, ^78^, ^79^, ^80^, ^81^, ^82^ This approach not only reduces the time required for cell reprogramming but also retains the epigenetic state of the biopsied cells, which are often reset during iPSC generation.^83^ Retaining these epigenetic features is particularly important for studying age‐related diseases, as it allows these induced neurons to reflect the aging phenotype of the donor.^82^ Recent work revealed that induced neurons express tau isoforms at both the transcript and protein levels, closely mirroring what is observed in the adult human brain.^79^ This makes such neurons valuable for investigating the onset and progression of neurodegenerative diseases.^79^, ^84^ Thus, our fibroblast resource offers a powerful platform for generating induced neurons to explore critical aspects of neurodegeneration within the context of African genetic backgrounds. Overall, our cell biobank sets a critical precedent for future biobanking efforts in Africa and serves as a critical resource for diverse fields requiring access to cells from African genetic backgrounds.

### 4.2 Genome editing to study tauopathies

In line with global efforts to develop disease‐specific iPSC models and our longstanding interest in tauopathies, we used CRISPR/Cas9–mediated genome editing to generate iPSC lines carrying well‐characterized *MAPT* mutations, P301L or R406W, which are linked to FTD.^59^, ^60^, ^61^, ^62^ These mutant lines retain key pluripotency markers and exhibit normal karyotypes, demonstrating their suitability for investigating tau‐related pathologies. While their intrinsic value for understanding disease mechanisms is significant, their utility is further enhanced compared to isogenic lines from other populations,^8^, ^11^, ^69^, ^70^, ^85^, ^86^ which can enable a comprehensive examination of ancestry‐related factors influencing the cellular mechanisms underlying tauopathies. Moreover, given the population‐level differences in *MAPT* haplotypes, this resource offers a unique opportunity to examine whether ancestry‐specific genetic variations modify tau expression, splicing, or aggregation in tauopathies. Thus, by integrating these edited lines into our biobank, we further extend the utility of this resource for researchers studying tauopathies and to support drug discovery pathways.

### 4.3 Global collaboration and distribution of iPSC lines

To address the absence of a local facility for generating and banking iPSCs in Nigeria, the BioRTC; www.BioRTC.com was established at Yobe State University, Nigeria, in 2021.^44^ This center, supported by the Yobe state government, now serves as the repository and distribution hub for the generated iPSC lines and their precursor somatic cells, with sub‐repositories established at existing biobanks at Washington University School of Medicine, USA, and the University of Sussex, UK. All 10 iPSC lines, the *MAPT*‐mutant isogenic pairs, and a previously generated iPSC line from an adult Nigerian donor^19^ have been banked at this newly established African Somatic and Stem Cell Bank.

Given that broad consent for global distribution was obtained from donors, these iPSC lines and derivatives are available to the global scientific community under a universal MTA. Our vision is that this resource—and additional lines currently in development—will provide a deeply characterized and genetically diverse collection of iPSCs derived from indigenous Africans. This will foster global collaboration, drive breakthroughs in the study of human diseases beyond neurodegenerative disorders, and accelerate drug discovery by enabling research in cells derived from African genetic backgrounds.

### 4.4 Concluding remarks

The African iPSC Initiative marks a transformative step in addressing the global underrepresentation of African populations in biomedical research. The iPSCs and their derivatives generated through this effort offer unparalleled potential for advancing our understanding of human diseases, particularly tauopathies, and for accelerating drug discovery in a historically underserved population. Future efforts aim to expand this resource by including iPSCs from a broader spectrum of African ethnic groups and generating disease‐specific lines, such as those for AD, to further support research on neurodegenerative and other diseases.

## CONFLICT OF INTEREST STATEMENT

The authors have no conflicts of interest to declare. Author disclosures are available in the supporting information.

## CONSENT STATEMENT

Written informed consent was obtained from all participants prior to sample collection. Consent included permission to derive, bank, and distribute iPSC lines for use in future research studies globally.

## Supporting information



Supporting Information

Supporting Information

Supporting Information

Supporting Information
