# Supplementary material for: Somatic and Stem Cell Bank to study the contribution of African ancestry to dementia: African iPSC Initiative
Source: Alzheimers Dement. 2025 Apr 12;21(4):e70145. doi: 10.1002/alz.70145 (PMC11992592; doi:10.1002/alz.70145)
Supplement: Supplementary file 2 — Supporting Information [file ALZ-21-e70145-s003.pdf]

Supplemental Figure 1

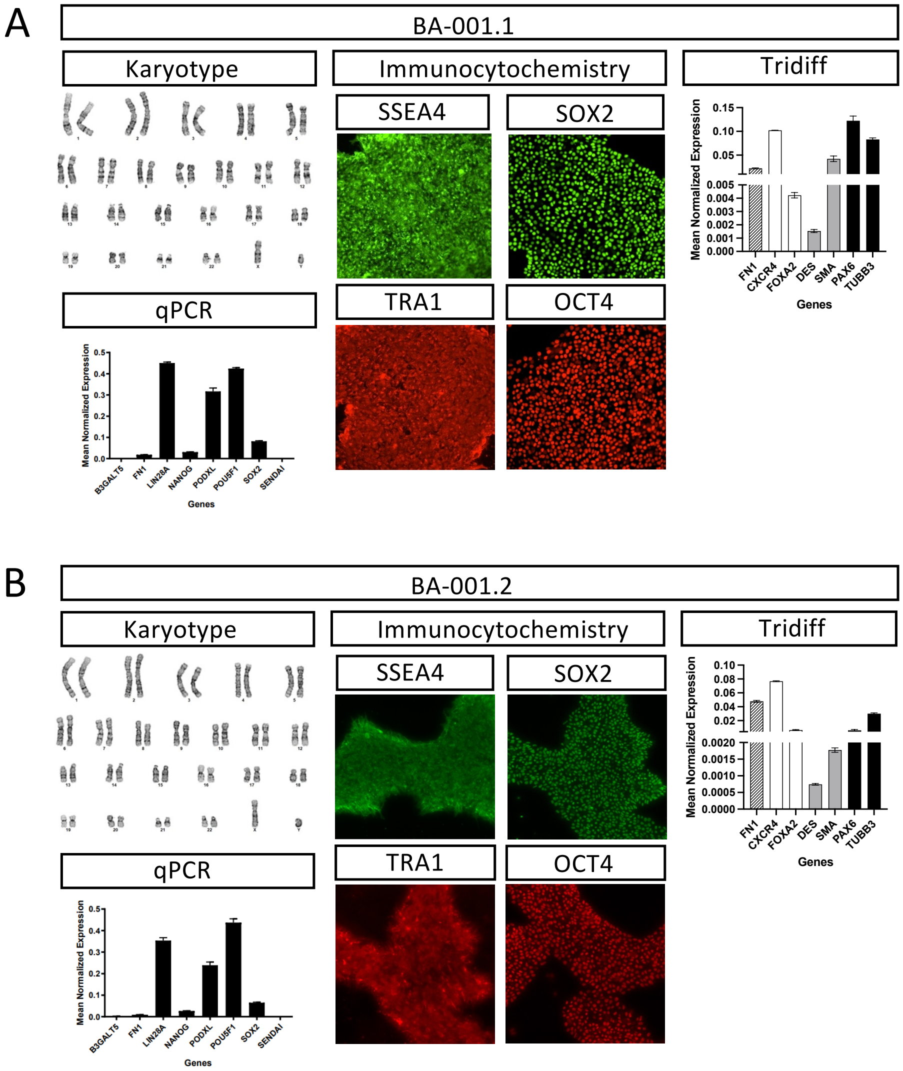

Supplemental Figure 1: Characterization of iPSC line BA-001. Representative iPSC characterization data. G-band karyotyping reveals no chromosomal abnormalities. qPCR for pluripotency markers normalized to GAPDH. Immunocytochemistry for pluripotency markers OCT4, SOX2, SSEA4, and TRA-1-60. Differentiation into cells found in each of the three germ layers were validated using qPCR for layer specific markers normalized to GAPDH. A. BA-001.1. B. BA-001.2.

Supplemental Figure 2

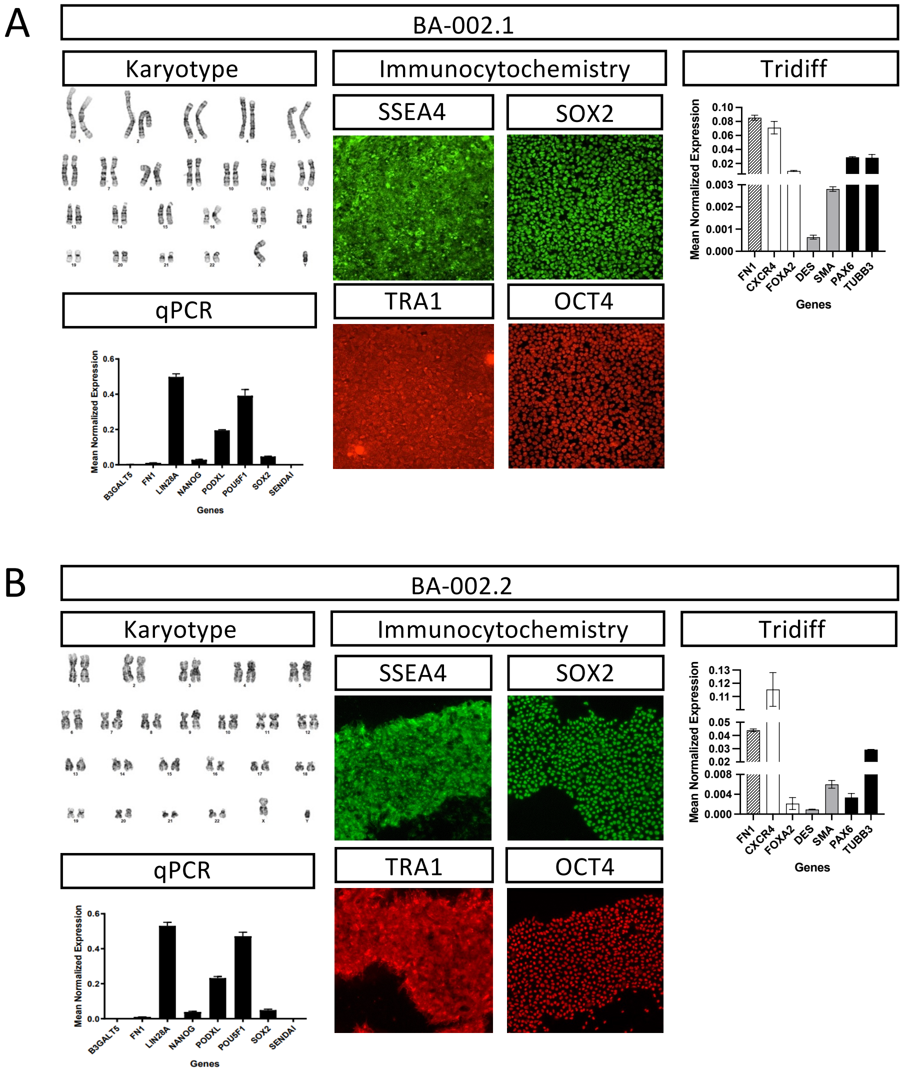

Supplemental Figure 2: Characterization of iPSC line BA-002. Representative iPSC characterization data. G-band karyotyping reveals no chromosomal abnormalities. qPCR for pluripotency markers normalized to GAPDH. Immunocytochemistry for pluripotency markers OCT4, SOX2, SSEA4, and TRA-1-60. Differentiation into cells found in each of the three germ layers were validated using qPCR for layer specific markers normalized to GAPDH. A. BA-002.1. B. BA-002.2.

Supplemental Figure 3

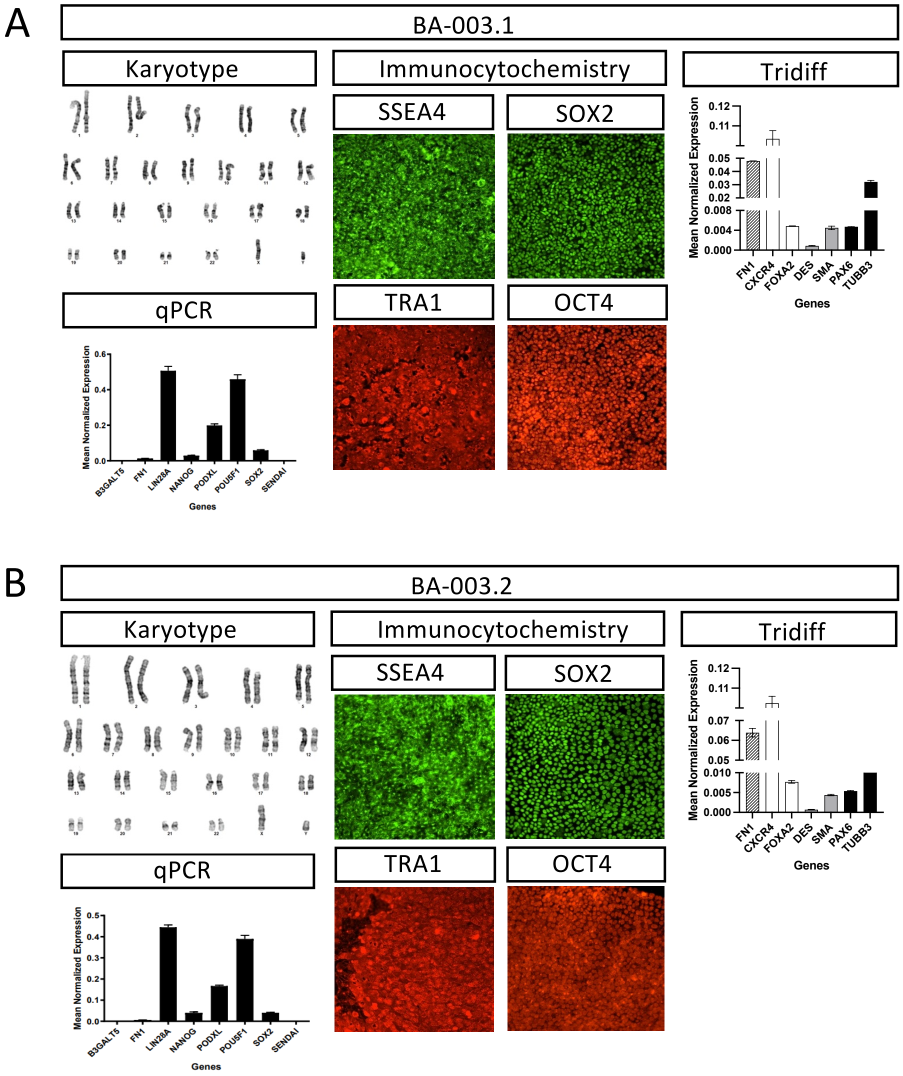

Supplemental Figure 3: Characterization of iPSC line BA-003. Representative iPSC characterization data. G-band karyotyping reveals no chromosomal abnormalities. qPCR for pluripotency markers normalized to GAPDH. Immunocytochemistry for pluripotency markers OCT4, SOX2, SSEA4, and TRA-1-60. Differentiation into cells found in each of the three germ layers were validated using qPCR for layer specific markers normalized to GAPDH. A. BA-003.1. B. BA-003.2.

Supplemental Figure 4

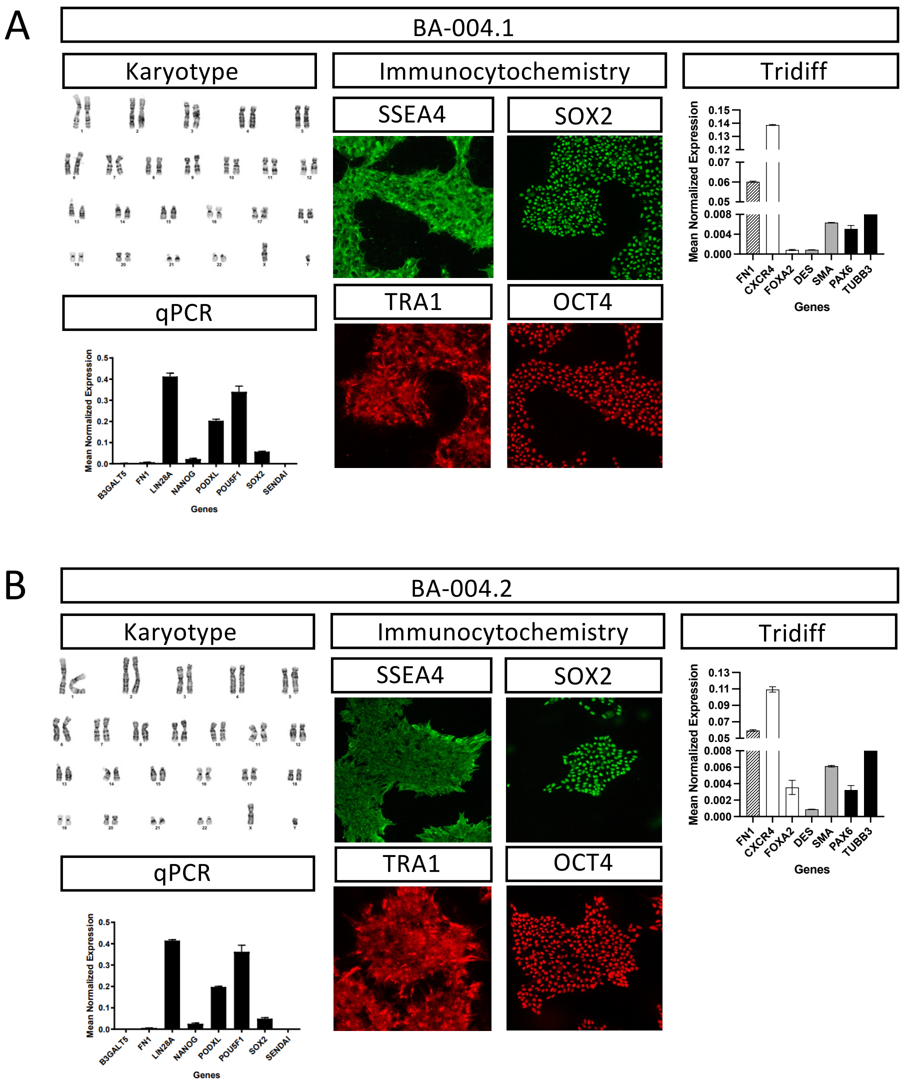

Supplemental Figure 4: Characterization of iPSC line BA-004. Representative iPSC characterization data. G-band karyotyping reveals no chromosomal abnormalities. qPCR for pluripotency markers normalized to GAPDH. Immunocytochemistry for pluripotency markers OCT4, SOX2, SSEA4, and TRA-1-60. Differentiation into cells found in each of the three germ layers were validated using qPCR for layer specific markers normalized to GAPDH. A. BA-004.1. B. BA-004.2.

Supplemental Figure 5

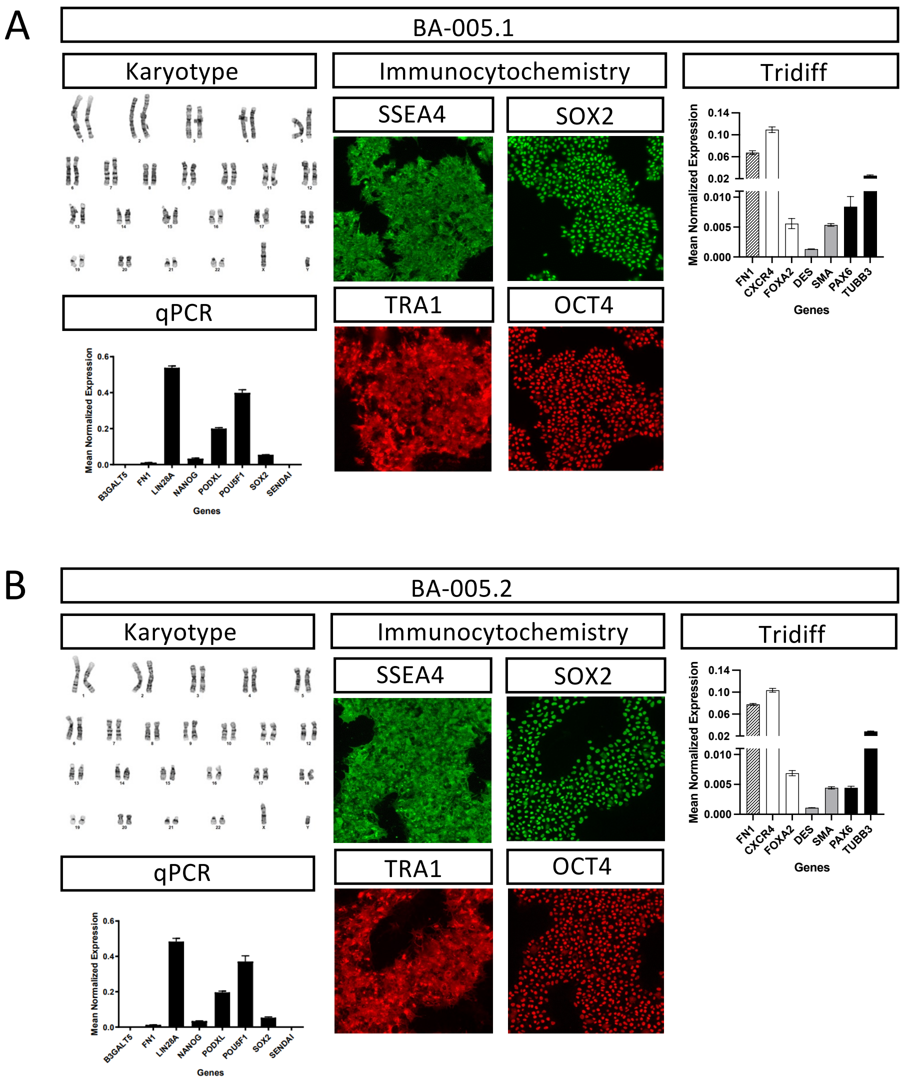

Supplemental Figure 5: Characterization of iPSC line BA-005. Representative iPSC characterization data. G-band karyotyping reveals no chromosomal abnormalities. qPCR for pluripotency markers normalized to GAPDH. Immunocytochemistry for pluripotency markers OCT4, SOX2, SSEA4, and TRA-1-60. Differentiation into cells found in each of the three germ layers were validated using qPCR for layer specific markers normalized to GAPDH. A. BA-005.1. B. BA-005.2.

Supplemental Figure 6

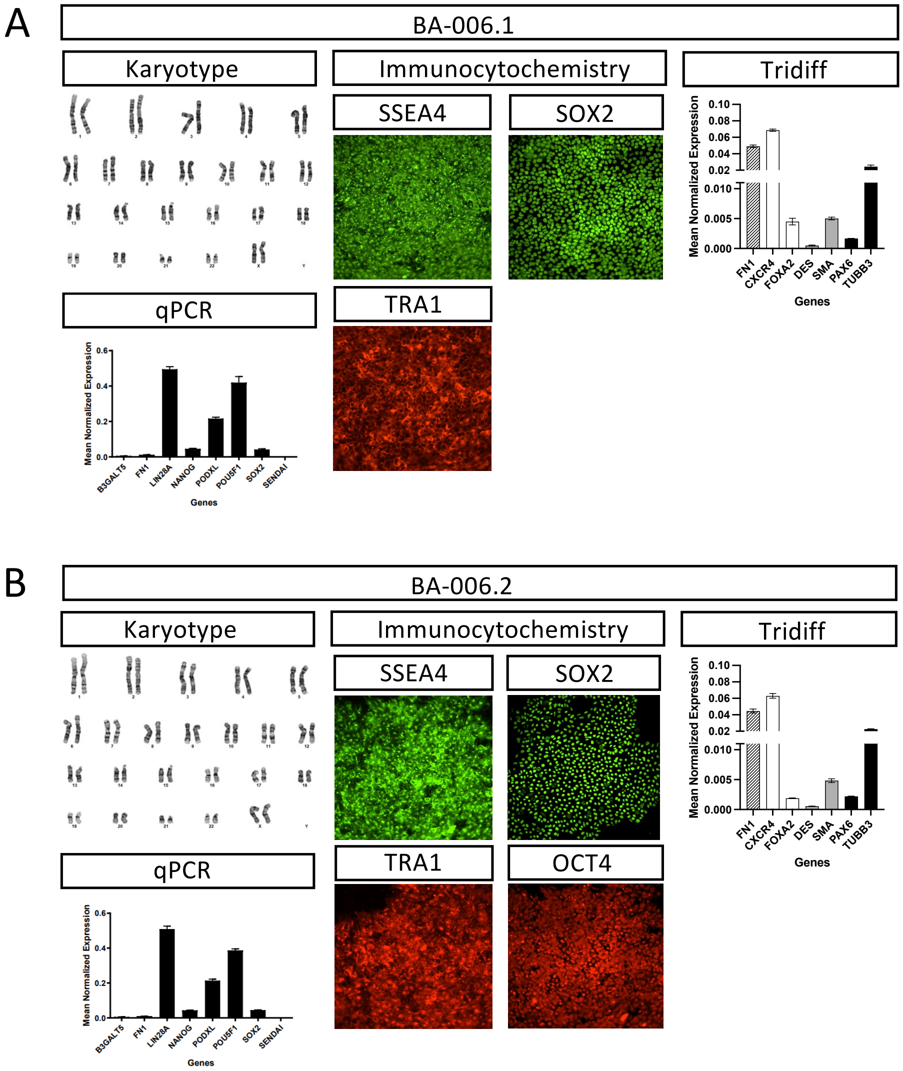

Supplemental Figure 6: Characterization of iPSC line BA-006. Representative iPSC characterization data. G-band karyotyping reveals no chromosomal abnormalities. qPCR for pluripotency markers normalized to GAPDH. Immunocytochemistry for pluripotency markers OCT4, SOX2, SSEA4, and TRA-1-60. Differentiation into cells found in each of the three germ layers were validated using qPCR for layer specific markers normalized to GAPDH. A. BA-006.1. B. BA-006.2.

Supplemental Figure 7

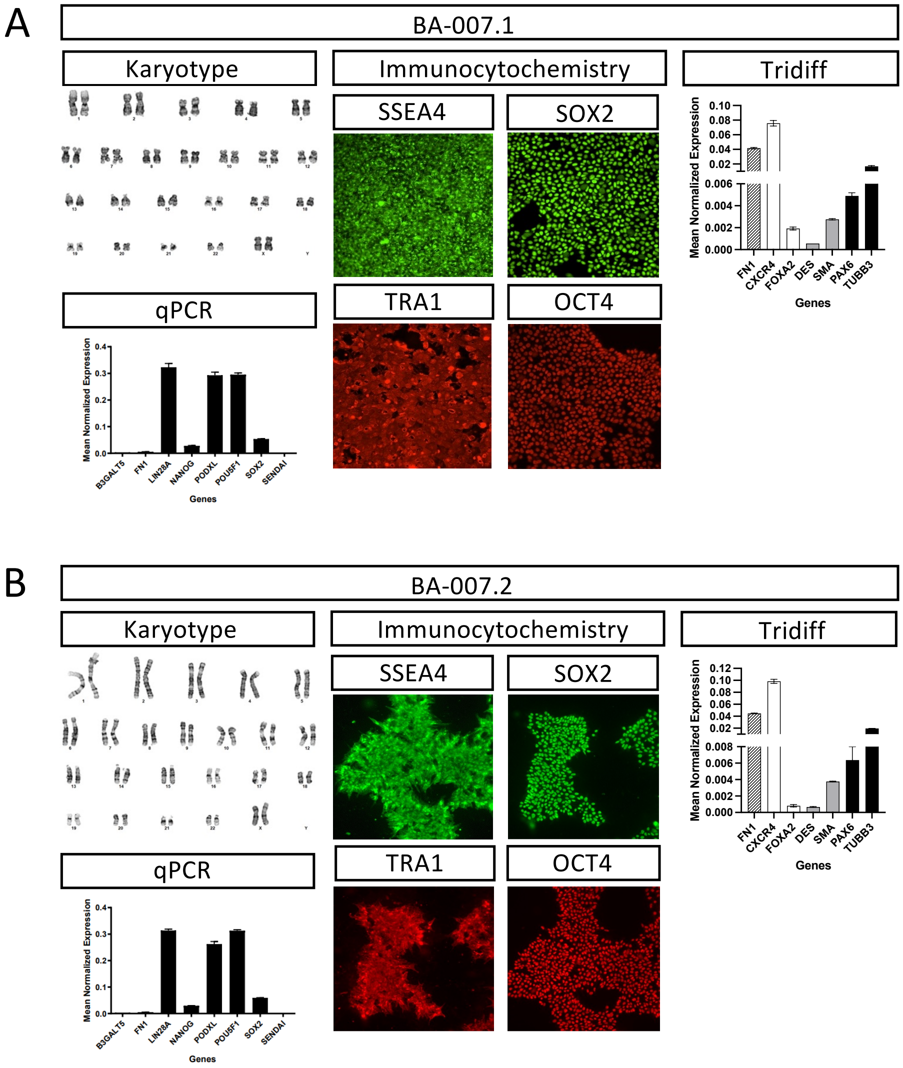

Supplemental Figure 7: Characterization of iPSC line BA-007. Representative iPSC characterization data. G-band karyotyping reveals no chromosomal abnormalities. qPCR for pluripotency markers normalized to GAPDH. Immunocytochemistry for pluripotency markers OCT4, SOX2, SSEA4, and TRA-1-60. Differentiation into cells found in each of the three germ layers were validated using qPCR for layer specific markers normalized to GAPDH. A. BA-007.1. B. BA-007.2.

Supplemental Figure 8

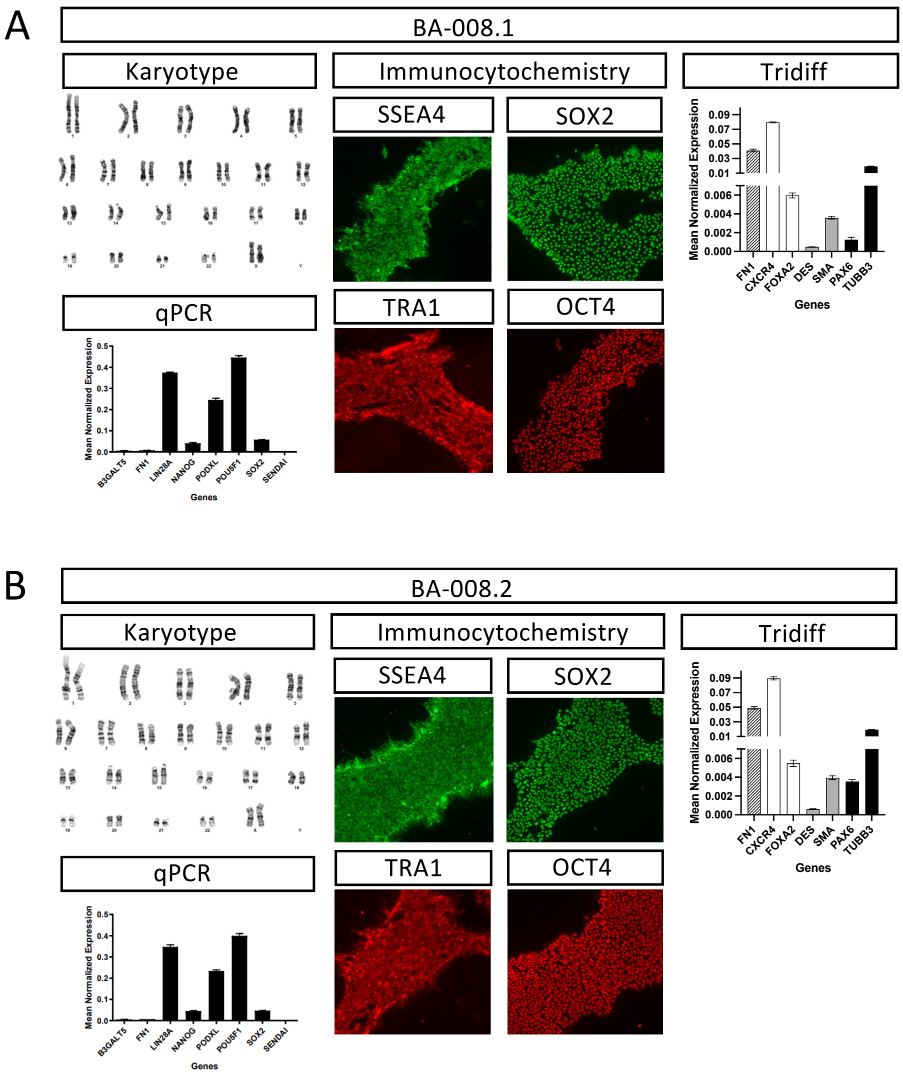

Supplemental Figure 8: Characterization of iPSC line BA-008.
Representative iPSC characterization data. G-band karyotyping reveals no chromosomal abnormalities. qPCR for pluripotency markers normalized to GAPDH. Immunocytochemistry for pluripotency markers OCT4, SOX2, SSEA4, and TRA-1-60. Differentiation into cells found in each of the three germ layers were validated using qPCR for layer specific markers normalized to GAPDH. A. BA-008.1. B. BA-008.2.

Supplemental Figure 9

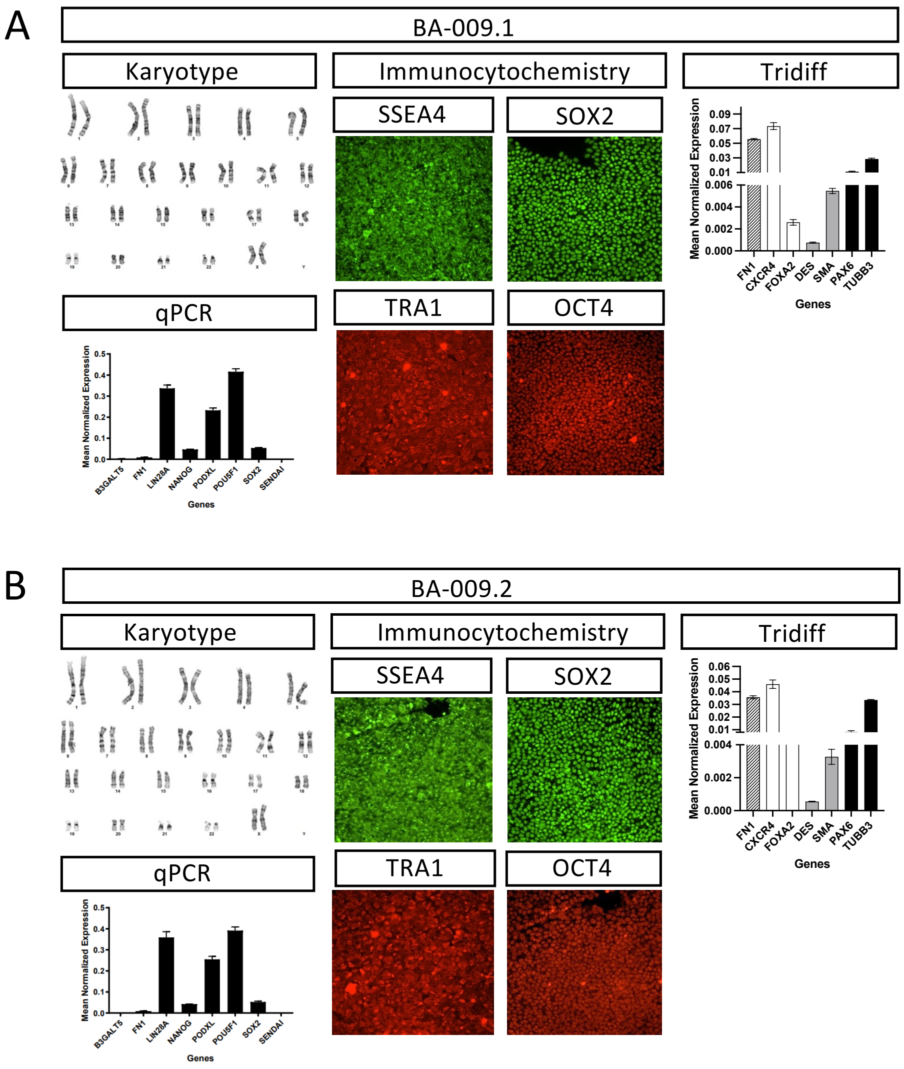

Supplemental Figure 9: Characterization of iPSC line BA-009. Representative iPSC characterization data. G-band karyotyping reveals no chromosomal abnormalities. qPCR for pluripotency markers normalized to GAPDH. Immunocytochemistry for pluripotency markers OCT4, SOX2, SSEA4, and TRA-1-60. Differentiation into cells found in each of the three germ layers were validated using qPCR for layer specific markers normalized to GAPDH. A. BA-009.1. B. BA-009.2.

Supplemental Figure 10

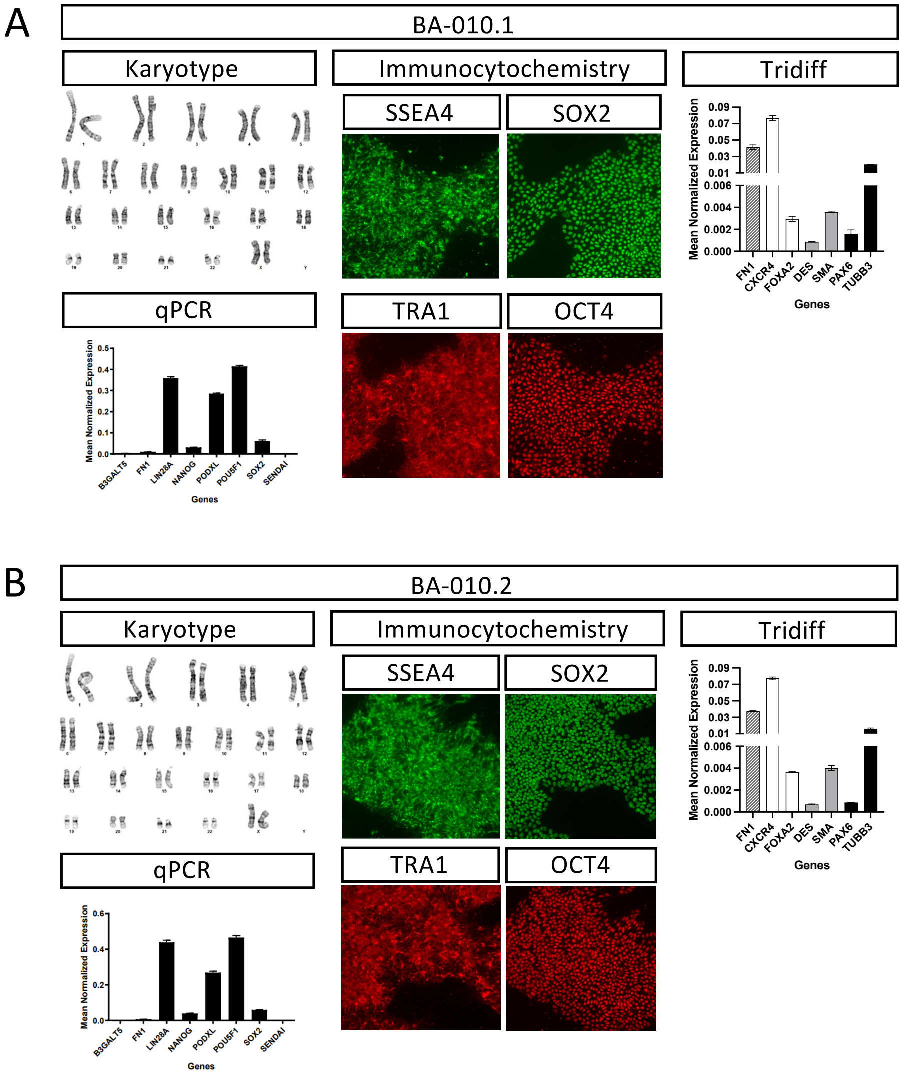

Supplemental Figure 10: Characterization of iPSC line BA-010. Representative iPSC characterization data. G-band karyotyping reveals no chromosomal abnormalities. qPCR for pluripotency markers normalized to GAPDH. Immunocytochemistry for pluripotency markers OCT4, SOX2, SSEA4, and TRA-1-60. Differentiation into cells found in each of the three germ layers were validated using qPCR for layer specific markers normalized to GAPDH. A. BA-010.1. B. BA-010.2.

Supplemental Figure 11

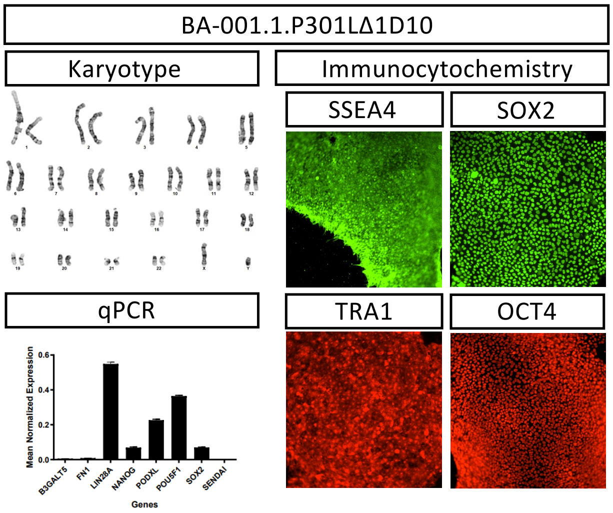

Supplemental Figure 11: Characterization of iPSC line BA-001.1.P301LΔ1D10. Representative iPSC characterization data. G-band karyotyping reveals no chromosomal abnormalities. qPCR for pluripotency markers normalized to GAPDH. Immunocytochemistry for pluripotency markers OCT4, SOX2, SSEA4, and TRA-1-60.

Supplemental Figure 12

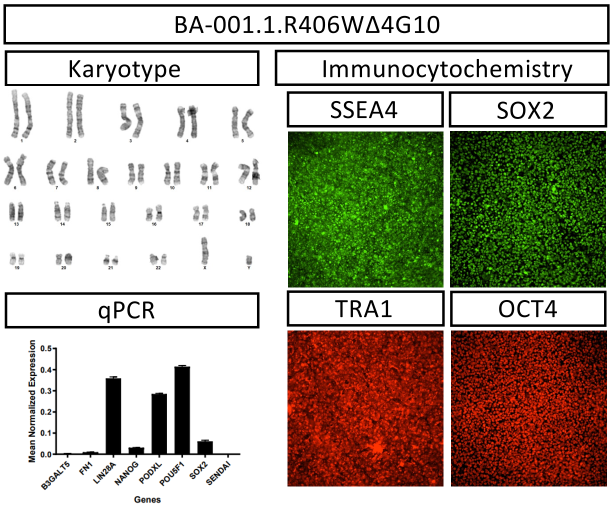

Supplemental Figure 12: Characterization of iPSC line BA-001.1.R406WΔ4G10. Representative iPSC characterization data. G-band karyotyping reveals no chromosomal abnormalities. qPCR for pluripotency markers normalized to GAPDH. Immunocytochemistry for pluripotency markers OCT4, SOX2, SSEA4, and TRA-1-60.
