# Supplementary material for: Somatic and Stem Cell Bank to study the contribution of African ancestry to dementia: African iPSC Initiative
Source: Alzheimers Dement. 2025 Apr 12;21(4):e70145. doi: 10.1002/alz.70145 (PMC11992592; doi:10.1002/alz.70145)
Supplement: Supplementary file 4 — Supporting Information [file ALZ-21-e70145-s002.pdf]

Informed Consent Form

Title of Study: Derivation of Human Induced Pluripotent Stem Cell Lines from an African-Resident Population for Neuroscience Research
Ethical Approval No.: YSUTH/MAC/EA/077/VOL.III/297
Sponsor/Funding: Alzheimer's Association
Principal Investigator: Mahmoud Bukar Maina
Institution: Biomedical Science Research and Training Centre (BioRTC), Yobe State University, Nigeria

Introduction

Good morning/afternoon. My name is ____, and I am a research assistant working with the Biomedical Science Research and Training Centre (BioRTC) at Yobe State University, Nigeria. We are conducting a study to generate human cells to help understand dementia and other neurodegenerative diseases. If you have an authorized representative or relative, they may sign this form on your behalf.

Purpose of the Study

The purpose of this study is to develop human cell lines that will help us better understand dementia and, potentially, contribute to treatment development. Currently, 60% of dementia cases occur in low- and middle-income countries, yet most biomedical research relies on cell samples from European populations. This study aims to address this disparity by establishing a registry of dementia and a bank of human cells derived from blood and skin cells of both healthy individuals and those with dementia. By participating in this research, you are contributing to efforts that could improve public health strategies for diagnosing and managing dementia and other neurological disorders.

Duration of the Study

This is a long-term study. You may be asked to participate on a time-to-time basis for as long as you are willing to take part or until the study is complete.

Procedures

If you agree to participate, you will:

- Visit a specified clinical site
- Undergo a physical examination
- Answer questions about your health history and that of your family, including any history of dementia or other neurological conditions
- Donate ~100ml (about 6 tablespoons) of blood
- Donate a small skin punch biopsy
- Have your caretaker interviewed (if applicable)

A trained healthcare professional will conduct these procedures, and you may be asked to revisit the clinic.
Data Use and Privacy

- Your biospecimens (e.g., blood, skin biopsy) and data will be used for this study and may be shared with other researchers locally or internationally for approved biomedical research.

- Samples may be used for future research, including disease modeling, drug development, and regenerative medicine.
- Your name and other identifying details will not be linked to your questionnaire responses or biological samples. Instead, all data will be coded and securely stored.
- Your study information is protected by a Certificate of Confidentiality, which allows researchers to refuse to share your information even in legal proceedings.
- Your data will be anonymized for research use.

Follow-up and Future Contact

- Your information has been securely recorded, and permission for follow-up has been obtained. This means we may contact you in the future for further studies or to assess your neurological health status.
- However, your participation remains voluntary, and you may decline further participation at any time.

Genetic Research and Whole Genome Sequencing

- This study involves genetic research, which may include whole-exome or whole-genome sequencing, to match the transformed cells to your original sample.
- Genetic information will be stored in a secure, controlled-access database.
- If research findings indicate you carry a gene variant linked to dementia or another neurodegenerative condition, these results would require confirmation through clinical testing before they can be shared with you.
- Some types of genetic analysis may reveal secondary findings unrelated to dementia. If such findings have potential health implications, you may be advised to seek clinical follow-up.
- Genetic research is ongoing, and notification of significant findings may take several years.

Commercial Use and Financial Considerations

- Your samples may be used in both academic and industry research.
- Research may contribute to the development of new therapies, but donors will not claim any intellectual property or financial benefit.
- You will not share in any commercial value or profits derived from your biospecimens, by-products, or data obtained from them.
- You will receive a small compensation for your participation, which is intended to cover travel expenses and lost work time.

Risks

- Other than anxiety, there are no anticipated risks associated with completing the questionnaire.
- You may experience mild pain and slight discomfort from the blood draw and skin biopsy.
- Excessive bleeding, bruising, fainting, or allergic reactions may occur in rare cases, particularly in individuals taking medication. A trained healthcare professional will be available to minimize these risks.

Benefits

- There is no direct benefit to you for participating in this study.
- However, your contribution may aid scientific discoveries that benefit future generations and improve dementia research, diagnostics, and treatments.

Right to Withdraw

- Your participation is entirely voluntary.
- You have the right to refuse to participate or withdraw from the study at any time without penalty.
- Refusal to participate will not impact your access to clinical care or medical services.
- If you choose to withdraw, any unprocessed samples will be destroyed. However, previously used samples or data that have already contributed to research may not be retrievable.

Confidentiality and Data Protection

- Your personal information will remain confidential.
- Data collected will be anonymized and stored securely.
- Your donated biosamples and derived cell lines may be made available to researchers worldwide under approved conditions.

Ethical Approval

- This study has received ethical approval from the Yobe State University Teaching Hospital Ethics Committee.
- Any future modifications to the study will require further ethical review.

Whom to Contact

If you have any questions about the study or your rights as a participant, you may contact:
Principal Investigator: Mahmoud Bukar Maina
Email: mahmoud.maina@biortc.com
Phone: +44 755 223 2355

Declaration of Consent

I have read and understood the information provided in this consent form. I have had the opportunity to ask questions and have received satisfactory answers. I voluntarily agree to participate in this study.

Name: ____
Sex: ___________
Ethnicity: ____
Date of Birth: ______________
Contact Phone Number: ______________
Educational Level: __________________

Do you have a health condition? ☐ Yes ☐ No
If Yes, what? _______________________

Signature of Participant: ______________________________ Date: ______________

If I become cognitively incapacitated, I authorize my next-of-kin to act on my behalf:

Authorized Representative Name: ____

Witness Name & Signature (if participant is unable to sign):
_______________________________________ Date: ______________

Confirmation of Consent (to be completed by data collector)

Recruitment Region: ____

I have confirmed with the donor that they have no further questions and wish to proceed with participation.

Signature of Data Collector: ______________________________________ Date: ______________
